# Careers in Disarray? COVID-19 and Self-Perceived Employability

**DOI:** 10.1177/10690727231187096

**Published:** 2023-06-27

**Authors:** Shuang Ren, Mohammad Tarikul Islam, Doren Chadee

**Affiliations:** 1 Queen’s Management School, 1596 Queen’s University Belfast, Belfast, UK; 2 School of Business Torrens University, Melbourne, VIC, Australia; 3 6939 Central Queensland University, Rockhampton, QLD, Australia

**Keywords:** COVID-19 pandemic, career shock, self-perceived employability, cognitive appraisal, career networking

## Abstract

The COVID-19 pandemic has produced disruptions to the employment and higher education contexts, exacerbating complexities involved in one’s assessment of their opportunities of employment in these contexts. The career literature has largely overlooked a vulnerable population of potential job candidates (i.e., final-year MBA students) who are at a critical juncture in response to COVID-19 career shock. Drawing from the challenge-hindrance appraisal framework, this research aims to theorize and test a moderated-mediation model in terms of how COVID-19 career shock associates with self-perceived employability. We use a simple random sampling procedure to collect data from 301 final year MBA students in Australia at the peak of the COVID-19 pandemic. The findings show that COVID-19 career shock can be perceived both as a challenge and a hindrance, which in turn associates with self-perceived employability differently. Results further demonstrate that the extent to which COVID-19 career shock is perceived as a challenge or hindrance is moderated by one’s career networking behavior. This research is a timely response to research calls for understanding how the COVID-19 has an impact on people’s work and career with a particular focus on a vulnerable yet under-studied group of labor force in the career literature.

The COVID-19 pandemic has substantially altered the employment prospects and career landscape of current and potential employees (Akkermans et al., 2020). Research is fast emerging on its effects for job occupants, exploring how organizations’ current employees navigate this unprecedented period of time. However, the experience of final-year MBA students during the pandemic, who constitute an important segment of future labor force, remains less-specified in the vocational field. From a career perspective, MBA students typically pursue higher education to further career growth (Wilkins et al., 2018). In their final year, they approach a critical juncture to make a personal self-assessment of employment possibilities appropriate to qualification level, termed self-perceived employability (Berntson & Marklund, 2007; Rothwell et al., 2009), which predicts important career outcomes, such as career success, career commitment and career satisfaction (De Vos et al., 2011). Research on determinants of self-perceived employability has unveiled the importance of human capital and social support (Wittekind et al., 2010). However, disruptions both in the higher education (e.g., Jung et al., 2021) and employment (Ng et al., 2021) contexts highlight an urgent need to advance a contextualized understanding of those MBA students’ self-perceived employability as a response to the COVID-19 pandemic.

In career terms, COVID-19 can be seen as a career shock, defined as “a disruptive and extraordinary event that is, at least to some degree, caused by factors outside the focal individual’s control and that triggers a deliberate thought process concerning one’s career” (Akkermans et al., 2018, p. 4). A recent survey shows that COVID-19 outbreak impacts university students’ current labor market participation and expectations about post-graduation labor outcomes, with working students suffering 31% decrease in wage, 37% drop in weekly working hours, and 40% loss of employment (Aucejo et al., 2020). Anecdotal evidence of student experience and expectations suggests that the COVID-19 career shock experience brought new challenges to the self-perceived employability domain.

While the COVID-19 as an economic and health crisis is stressful, the value of approaching COVID-19 experience as a career shock is that this emerging literature does not narrowly portray career shock as bleak only (Akkermans et al., 2020). The most recent debate on the topic suggests that career shock experience could result in positively valenced career opportunities in a longer time-frame (Ng et al., 2021). It brings an unintended reflection on work, life and future in relation to opportunities for growth (Hite & McDonald, 2020). The renewed recognition of building a sustainable post-COVID-19 career and investing in ways to navigate through the rise of job insecurity and precarious work points to the importance of resources that people build and preserve (Hobfoll, 1989). Research suggests that people weigh their personal resources and external factors in estimating their own employability (Coetzee & Engelbrecht, 2020; Creed & Gagliardi, 2015; Rothwell et al., 2009).

However, based on the transactional theory of stress (Lazarus & Folkman, 1984), it is not just the availability of personal resources per se that determines one’s coping, but framing the meaning of an event in relation to oneself that directs the subsequent attitudes and behavior. In other words, individuals appraise the extent to which they can access and mobilize personal resources to address the contextual career demands caused by COVID-19, which is manifested in their appraisal of COVID-19 as challenging or hindering. While the career shock literature has drawn from career self-management theory (Seibert et al., 2013) or conservation of resources theory (Hobfoll, 1989) to explain its possible impact, how it influences the individual appraisal process, including the subsequent consequence, remains under-theorized and tested. This limitation risks overlooking the cognitive and behavioral responses that individuals use to manage an external event vis-a-vis their personal resources.

The aim of this research is to address the limitation by examining how and when the COVID-19 career shock associates with an individual’s self-perceived employability. We draw from the transactional theory of stress (Lazarus & Folkman, 1984) to propose a fuller picture that the COVID-19 career shock can be perceived as both a challenge appraisal that offers opportunities for growth and a hindrance appraisal that hinders goal pursuit, which in turn associates with self-perceived employability differently. Further, given the importance of an assessment of coping resources, we further argue that career networking behavior (developing and maintaining relationships with others who have the potential to assist careers (Forret & Dougherty, 2004) moderates the relationships under study. The study makes three main contributions. First, it is a timely response to research calls for understanding how the COVID-19 influences career with a particular focus on a vulnerable yet under-studied group of labor force (see Akkermans et al., 2020). Second, it clarifies the underlying mechanism through which the implications of COVID-19 career shocks are translated into self-perceived employability. By so doing, it enriches the theoretical development of the career shock literature and offers a nuanced theorization and empirical evidence that the COVID-19 career shock can be perceived as providing opportunities for growth and for impairment. Third, it introduces a boundary condition (i.e., career networking behavior) regarding the relationship between career shock, challenge/hindrance appraisal, and self-perceived employability.

## Theoretical Framework

Transactional theory of stress explains people’s behavior through the way people interpret the encountered events, which influences subsequent coping strategy (Lazarus & Folkman, 1984). A challenge appraisal is formed when the event is perceived to promote goal pursuit and hence promotes positive emotions and problem-focused coping (Searle & Auton, 2015). A hindrance appraisal is formed when the event is perceived to cause potential harm/loss, inducing negative emotions and avoidant coping strategies (Searle & Auton, 2015). The value of unfolding the appraisal process versus a priori categorization of events into challenge/hindrance stressors is that a stressor can be interpreted in different ways (Hobfoll, 1989). Consistent with Lazarus (1991), empirical research has shown that challenge and hindrance appraisals are two independent cognitive appraisal that can co-exist, rather than being mutually-exclusive (Gerich & Weber, 2020; Prem et al., 2017; Webster et al., 2011). For instance, the cognitive and affective characteristics of the coping efforts differ – challenge appraisal orients towards the opportunity for achieving potential gains, with an expectation of successful coping, whereas hindrance appraisal orients towards avoiding unfavorable outcomes, anticipating loss (Folkman et al., 1986; Tomaka et al., 1993). As Hobfoll (1989) put it, an event is seldom clearly positive or negative, but is subjective to the evaluation.

### Hypothesis Development

According to the transactional theory of stress (Lazarus & Folkman, 1984), not all events trigger the appraisal process – it occurs when the events introduce the potential for growth/gain or loss/harm. The COVID-19 as a career shock is likely to prompt a challenge appraisal for two main reasons. First, the COVID-19 career shock interrupts the status quo and highlights a need to reconsider alternative opportunities and innovative ways to address the problem. For instance, research has observed that the COVID-19 accelerates the telecommuting arrangement of work. The increasing trend of automation and the likely persistent use of digitalization point to areas of upskilling or re-skilling that have short and long-term benefits for employability (Hite & McDonald, 2020). The motivation for upskilling or reskilling provides individuals with solution-oriented outlook for future improvements.

Second, the COVID-19 career shock is an extraordinary event, high in intensity and duration (Akkermans et al., 2020). The job loss, layoffs and high unemployment rate are largely due to the measures taken worldwide to curb the pandemic, such as travel bans, border closure, quarantine measures and lock-downs, causing significant declines in revenue and insolvencies in the related sectors (Rivera, 2020). Thus, it may not be taken personally. In other words, individuals who experience a career shock as a result of COVID-19 are less likely to see it reflecting their personal inadequacy, but more as seeing themselves being vulnerable to labor market disruptions due to economic impacts of the pandemic. Research has shown that attributing negative experience to external changing factors provides people with hope that the future may be different from the past (Weiner, 2010). Therefore, we argue:

Hypothesis 1:
COVID-19 career shock is positively associated with challenge appraisal.

In the meantime, the COVID-19 career shock is unpredictable and the stress literature has suggested that the lack of controllability impairs coping abilities and induces depression (Lucas et al., 2014). Research shows that a hindrance appraisal occurs when it is difficult to determine whether an investment of time and energy will be rewarded (Cavanaugh et al., 2000). Indeed, those who have stopped working as a result of the COVID-19 have reported increasing distress and declining mental and physical health conditions (Zhang et al., 2020). Further, an emerging consensus that some jobs or sectors will inevitably disappear in the aftermath of the COVID-19 (Hite & McDonald, 2020) means people in these jobs or sectors will be unlike to have employment opportunities even though they are capable of doing the job. In this sense, the disruptions embedded in the COVID-19 career shock may be perceived by not offering the opportunities for growth and thwarting gains in personal wellbeing and welfare (Cavanaugh et al., 2000). Therefore, we argue that the COVID-19 career shock can be appraised by people as a hindrance that constrains goal achievement, growth and wellbeing.

Hypothesis 2:
COVID-19 career shock is positively associated with hindrance appraisal.

We next argue that challenge appraisal of COVID-19 career shock in turn can be positively associated with self-perceived employability. From a transactional theory of stress perspective (Lazarus & Folkman, 1984), this positive association is due to three explanations that are consistent with prior work on graduate employability (Homes, 2013). First, challenge appraisal focuses on the potential growth in an event and stimulates positive affect, which makes individuals motivated to engage in skill building and learning (Shirom, 2003; Warr et al., 2014). This helps people to enhance their knowledge and skills needed for employment. Second, when an external event is perceived as challenging but manageable (i.e., a challenge appraisal), people are motivated to increase their efforts towards goal pursuits (Perrewé & Zellars, 1999), which may manifest in working towards a position where desirable jobs are offered. Third, the enhanced efforts may also be manifested in the investment of time and energy to present themselves more appropriately or attractively to employers, which enhances their perceived chance of obtaining employment. Indeed prior research on the antecedents of self-perceived employability has noted that positive cognitive and emotional processes are likely to enhance an individual’s perception of their capacity for enhancing employment (Álvarez-González et al., 2017). Therefore, we hypothesize:

Hypothesis 3:
Challenge appraisal is positively associated with self-perceived employability.

Using a similar line of logic, we expect a hindrance appraisal of COVID-19 career shock will be negatively associated with self-perceived employability. Specifically, a hindrance appraisal activates negative affect by seeing themselves hindered from pursuing self-relevant goals (Rodell & Judge, 2009). Negative affect leads to low-effort cognition (Sullivan & Conway, 1989), and reduced task performance (Gillet et al., 2013), which are not conducive to the possession of KSAs needed for employability. The perceived gap between their employability and the demands of changing labor market as a result of the COVID-19 may hence result in a lower level of self-perceived employability. In addition, a hindrance appraisal promotes negative emotion-focused coping behavior (Mackey & Perrewe, 2014). When this occurs, people tend to distance themselves from the situation by showing withdrawal behavior and/or reduced motivation (Perrewé & Zellars, 1999). Also, without motivation, people are unlikely to engage in developing and presenting themselves to employers to enhance their employability.

Hypothesis 4:
Hindrance appraisal is negatively associated with self-perceived employability.

Integrating the reasoning above, we further propose a mediating relationship below:

Hypothesis 5:
Challenge appraisal mediates the relationship between COVID-19 career shock and self-perceived employability.

Hypothesis 6:
Hindrance appraisal mediates the relationship between COVID-19 career shock and self-perceived employability.

### The Moderating Role of Career Networking Behavior

Based on the transactional theory of stress, the way people appraise an event is influenced by their assessment of the controllability and coping potential (Folkman et al., 1986). This suggests behaviors that enable individuals to believe in better control of the condition or coping with the stressor are likely to influence the magnitude of how the COVID-19 career shock is appraised. To this end we argue that career networking behavior, the intentional pursuit of professional relationships for career development purposes (Seibert et al., 2013), serves as an important career-related resource that moderates the implications of career shock.

Specifically, research has shown that career networking behavior provides people with access to valuable career resources such as social capital, career advice, job opportunities and positive career outcomes (Spurk et al., 2015), which can enable them to better control their career experiences. For instance, career networking motivates proactive skill development in which people actively read or attend courses for knowledge and skill acquisition and contact development (Ren & Chadee, 2017). So, when people score higher in career networking behavior, they are more likely to amplify the KSA base. In face of the COVID-19 career shock, the amplified KSA base can lead to a more positive assessment of possibilities of employment. Also by actively expanding the basis and quality of social contacts for career benefits, career networking behavior enables positive affect (Volmer & Wolff, 2018), which broadens thought-action repertoires to view the context in a more positive outlook. Therefore as a proactive resource management strategy, career networking behavior provides a range of additional coping resources that people can use to make a more forward thinking, positive evaluation of their employment. By contrast, for people scored lower in career networking behavior, they do not have the same level of access to these career resources compared to those scored higher. Without additional resources obtained through networking, people are less likely to believe the COVID-19 career shock is under their control. Therefore we hypothesize below:

Hypothesis 7:
Career networking behavior positively moderates the relationship between COVID-19 career shock and challenge appraisal such that the relationship is stronger (weaker) when career networking behavior is high (low).

Along the similar line of reasoning, career networking behavior expresses the individual’s investment in developing important resources (e.g., social capital, occupational expertise, and career support (Hirschi & Koen, 2021; Spurk et al., 2015) to navigate the career landscape. Through the career network, advice or opportunities obtained from a higher (vs. lower) level of career networking behavior, people are more likely to gain better insights into how a particular career shock is affecting their (future) employment. These insights provide guidance for people in terms of how or where to acquire coping resources to reduce the negative impact of such career shock. In this way, when people engage in higher career networking behavior, they are far less likely to see the career shock as thwarting their career pursuit. By contrast, when proactive networking behavior is lower, people have less access to career-relevant resources, either in the form of KSAs directly (e.g., knowledge acquisition) or indirectly (e.g., developmental opportunities, career insights and career support). When this occurs, in evaluating their resources against the demands in the external environment, people are more likely to perceive the COVID-19 career shock as threatening and feeling they are less capable of coping with it. Therefore, we propose:

Hypothesis 8:
Career networking behavior negatively moderates the relationship between COVID-19 career shock and hindrance appraisal such that the relationship is weaker (stronger) when career networking behavior is high (low).

### The Current Study

**Figure 1.**
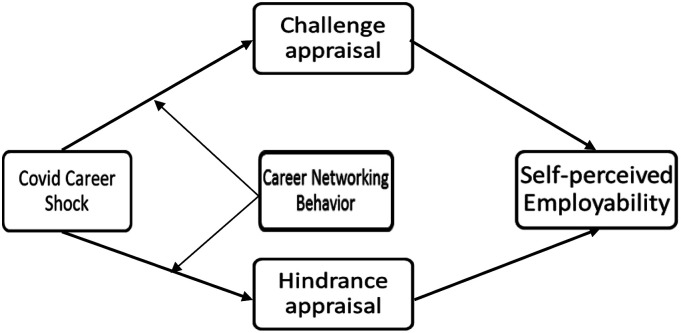
Conceptual framework.

This study examines the association between COVID-19 career shock and appraisals (H1: challenge appraisal; H2: hindrance appraisal) and the association between appraisals and self-perceived employability (H3: challenge appraisal; H4: hindrance appraisal), which in turn we propose a mediating relationship between COVID-19 career shock and self-perceived employability via challenge (H5) and hindrance appraisals (H6) accordingly. Finally, we propose that career networking behavior moderates the relationship between COVID-19 career shock and challenge (H7) and hindrance (H8) appraisals respectively (Figure 1).

This study uses data collected in two waves between September and December 2020, which is the end of an academic year and at the peak of the pandemic in Australia. Australia adopted strict and long restrictions to manage the pandemic, including border closures and protracted lockdowns, which resulted in classes moving online and unemployment rate soared (Stobart & Duckett, 2022). We highlight the pandemic experience of MBA students because they are not only the future skilled labor force but also contribute substantially to the part-time and casual labor force while undertaking their studies.

## Methods

### Participants and Procedure

Participants are final year MBA students enrolled at a major university in Australia. A simple random sampling approach was used in which a total of 1100 students were randomly selected, with an email invitation describing the purpose of the research sent a week before the distribution of the survey. The first wave comprised questions related to COVID-19 career shock, career networking behavior, challenge and hindrance appraisals and demographic variables. After three email reminders at one-week interval to non-respondents, a total of 420 responses were received by the cut-off date. The second wave survey sought responses on self-perceived employability, from the 420 respondents who completed the first wave survey. 386 responses were received following three email reminders at one-week interval. After data cleaning that excluded incomplete responses and speeders (completed the survey in less than the median completion time), we retained 301 responses for analysis. A time-separated design helps to optimize cross-sectional research (e.g., Seol et al., 2023). Following Rogelberg and Stanton’s (2007) recommendations, we conducted a wave analysis comparing the early 100 respondents and the last 100 respondents. Independent sample *t* test showed no significant differences across the main variables between the two groups, which indicates that non-response bias did not present a major issue with our data.

The final sample comprised 55.1% women, 55.1% single and 28.9% married without children. The majority was less than 30 years old (58.3%) and 31–40 years old (34.7%). All participants were working when commencing their MBA programs. At the time when the survey was conducted, 64.1% were in part-time employment while 35.9% had recently lost their jobs due to the COVID-19. The majority (58%) had 1–3 years of full-time equivalent work experience, followed by 25% with more than 6 years’ experience.

### Measures

All measures were based on existing scales and assessed on a seven-point Likert scale (1 = not at all; 7 = to a very large extent) unless indicated otherwise. We followed the three-stage procedure recommended in the literature (Islam & Polonsky, 2020) to ensure validity of the measures, including: (i) expert review (*n* = 5) of academics with subject expertise; (ii) an in-depth interview (*n* = 10) to ensure the questions were clearly understood and reflected participants’ experience to the COVID exposure; and (iii) a pilot study of the surveys (*n* = 30).

Career shock COVID-19 career shock was measured using four items adapted from Seibert et al. (2013) career shock scale, which have been used globally in the literature (e.g., Blokker et al., 2019; Spurk et al., 2015). Respondents were asked to indicate to what extent the COVID-19 pandemic impacted their career-related events that influence their career path or career decision making. Sample item includes: “I have a mentor or colleague that was important to me leave the company due to COVID-19” (1 _ have not experienced it; 2_had minimal impact; 3_had some impact; 4 _ had small impact; 5 _ had moderate impact, 6_had large impact and 7_had extremely large impact). Cronbach’s alpha was .83.

Career networking behavior Career networking behavior was measured using five items from Sturges et al., (2002), which are also used by De Vos et al. (2009) and De Vos & Soens (2008) on student samples. Sample item includes: “I have got myself introduced to people who can influence my career”. Cronbach’s alpha was .87.

Challenge appraisal Challenge appraisal was measured using three items from Lazarus and Folkman (1984) which are widely used (e.g., Lechner et al., 2016; Ma et al., 2021). Respondents indicated the extent to which they perceived the COVID-19 pandemic promoted personal growth, goals and accomplishments (LePine et al., 2005). Sample item includes: “Helped you improve your personal growth and well-being”. Cronbach’s alpha was .82.

Hindrance appraisal Hindrance appraisal was measured using three items from Lazarus and Folkman (1984), which are widely used (e.g., Lechner et al., 2016). Participants indicated the extent to which they perceived the COVID-19 pandemic thwarted personal goal accomplishment, growth and learning (LePine et al., 2005). Sample item includes: “Obstructed your personal growth and well-being”. Cronbach’s alpha was .91.

Self-perceived employability Self-perceived employability was measured using five items from Berntson and Marklund (2007), which is frequently used (Harari et al., 2021), including on university student samples (e.g., Chiesa et al., 2020). Sample item includes: “My competence is sought-after in the labor market”. Cronbach’s alpha was .86.

Controls Guided by past research (Blokker et al., 2019) on career shock and perceived employability, age (coded as 0 = <30 years; 1 = 31–40 years; 2 = 41–50 years; and 3 = >50 years), gender (coded as 0 = male; 1 = female; and 2 = prefer not to disclose), work experience (measured as 0 = <1 year; 1 = 1–3 years; 2 = 4–6 years; and 3 = >6 years), marital status (coded as 0 = single; 1 = married without children; and 2 = married with children) and current employment status (coded as 0 = unemployed; 1 = employed; and 2 = self-employed) are included in our model to control for their influences on the relationships under study.

### Data Analysis

SPSS PROCESS macro^1^ v3.5.3 (Hayes, 2013) is used given its capability of assessing moderation and mediation simultaneously. Mean-centred technique was used to address multicollinearity in moderation analysis (Marsh et al., 2004). We use bootstrapping analysis to obtain 95% bias-corrected bootstrapping confidence intervals, based on 5,000 resampling.

## Results

### Descriptive Statistics

Table 1 summarizes the mean, standard deviation and intercorrelations of all variables used in our study. A series of confirmatory factor analysis showed the hypothesized five-factor model fit the data well (Chi-square = 201.583, df = 158, CFI = .984, TLI = .981, RMSEA = .030, SRMR = .053) and performed better than alternative models, including a four-factor model where hindrance appraisal and challenge appraisal combined into one factor (Chi-square = 817.539, df = 164, CFI = .760, TLI = .722, RMSEA = .115, SRMR = .111), a two-factor model where COVID-19 career shock, career networking behavior, challenge appraisal and hindrance appraisal combined into one factor (Chi-square = 1788.698, df = 169, CFI = .405, TLI = .332, RMSEA = .179, SRMR = .184), and a one-factor model (Chi-square = 2135.281, df = 170, CFI = .279, TLI = .194, RMSEA = .196, SRMR = .204).

**Table 1.** Descriptive Statistics of Study Variables.

Variables	Mean	SD	1	2	3	4	5	6	7	8	9	10
1 COVID-19 career shock	4.24	1.56	***.79(.83)***									
2 Career networking behavior	4.22	1.48	.29**	***.82(.87)***								
3 Challenge appraisal	4.11	1.54	.27**	.27**	***.80(.82)***							
4 Hindrance appraisal	4.06	1.63	.25**	−.17**	−19**	***.84(.91)***						
5 Self-perceived employability	4.30	1.15	.23**	.31**	.27**	.25**	***.82(.86)***					
6 Age	.49	.63	−.18**	−.37**	.25**	−.22**	.17**	---				
7 Gender	0.56	.50	.13*	−.13*	−.21**	.18**	.14*	.14*	---			
8 Work experience	1.41	1.14	.17**	.21**	.19**	.24**	−.27**	.53**	.09 *n.s*.	---		
9 Marital status	.61	.75	−.25**	−.08 *n.s*.	−.17**	.18**	.19**	.38**	.18**	.27**	---	
10 Current employment status	.75	.64	.20**	−.14*	−.18**	.17**	.18**	−.10 *n.s.*	−.06 *n.s.*	.18**	.12*	---

*Notes*. *n* = 301. The square root of the average variance extracted of each construct along the diagonal line and the corresponding Cronbach’s alpha within the bracket. Age (measured as 0 = <30 years; 1 = 31–40 years; 2 = 41–50 years; and 3 = >50 years), gender (coded as 0 = male; 1 = female; and 2 = prefer not to disclose), work experience (measured as 0 = <1 year; 1 = 1–3 years; 2 = 4–6 years; and 3 = >6 years) and marital status (coded as 0 = single; 1 = married without children; and 2 = married with children), employment status (coded as 0 = unemployed; 1 = employed; and 2 = self-employed). The bold and italicized values are Cronbach's Alpha and the square root of the average variance extracted.

**p < .01; *p < .05.

### Hypotheses Testing

Results of hypothesis testing are summarized in Table 2. Hypotheses 1 and 2 posit COVID-19 career shock positively associates with challenge appraisal and hindrance appraisal respectively. We used model 4 in PROCESS macro for this simple mediation estimation to construct the indirect effect. The significant association between COVID-19 career shock and challenge appraisal (b = .390, SE = .104, *p* = .000) and hindrance appraisal (b = .738, SE = .127, *p* = .000) provides support for both H1 and H2. The significant result of the association between challenge appraisal and self-perceived employability (b = .583, SE = .107, *p* = .000) supports H3. Hypothesis 4, which predicts a negative association between hindrance appraisal and self-perceived employability, was supported (b = −.470, SE = .077, *p* = .000). As shown in Table 3, Hypotheses 5 and 6, which posit that challenge appraisal (indirect effect = .227, SE = 072, 95% CI = .104 to .238) and hindrance appraisal (indirect effect = −.347, SE = 089, 95% CI = .168 to .409) mediate the association between COVID-19 career shock and self-perceived employability respectively, are also supported.

**Table 2.** Unstandardized Regression Results.

	Challenge Appraisal			Hindrance Appraisal			Self-Perceived Employability		
	*b* (se)	*p*	95% C.I.	*b* (se)	*p*	95% C.I.	*b* (se)	*p*	95% C.I.
Control variables									
Age	.621(.153)	.000	.385–1.626	.693 (.21)	.000	.385–1.626	1.700(.456)	.000	.389–3.011
Gender	.254(.081)	.015	.783–1.291	.155 (.051)	.031	.907–1.217	1.597(.416)	.000	.250–2.944
Work experience	.247(.062)	.017	.299–.783	.504 (.219)	.032	.145–1.053	−.236(.131)	.034	.466–.938
Marital status	−.852(.379)	.000	.105–1.598	.583 (.231)	.000	.182–1.347	.221(.103)	.041	.631–.984
Current employment status	.599(.105)	.000	.189–1.387	−.329 (.101)	.005	.478–1.136	.667(.235)	.000	.960–1.093
Predictors									
COVID-19 career shock (CS)	.390(.104)	.000	.070–.364	.738 (.127)	.000	.195–382	.031(.061)	.625	−.090–.151
Career networking behavior (CN)	.164(.041)	.000	.084–.244	−.096 (.042)	.022	−.178-(-014)	.032(.050)	.526	−.067–.131
CS * CN	.331(.060)	.000	.167–.377	−.291 (.068)	.001	.153–.294	−.005(.007)	.527	−.017–.009
Mediators									
Challenge appraisal							.583(.107)	.000	.251–.487
Hindrance appraisal							−.470(.077)	.000	.186–.383
*R square*	*.291*			*.421*			*.323*		

*Note*. *n* = 301; se = Standard Error; C.I. = Confidence Interval.

**Table 3.** Results of Conditional Indirect Effects.

	*b*	se	95% C.I.
Mediated relationships			
CS-Challenge appraisal-self-perceived employability	.227	.072	.104–.238
CS-Hindrance appraisal-self-perceived employability	−.347	.089	.168–.409
Moderation Mediation			
Index of moderated mediation via challenge appraisal	.050	.031	.158–.627
Conditional indirect effect at high and low levels of CN through challenge appraisal High CN (+SD)	.243	.078	.110–.254
Low CN (-SD)	.207	.059	.101–.223
Index of moderated mediation via hindrance appraisal	−.032	.021	.081–.323
Conditional indirect effect at high and low levels of CN through hindrance appraisal High CN (+SD)	−.358	.094	.176–.435
Low CN (-SD)	−.332	.074	.159–.378

*Note*: *n* = 301; se = Standard Error. CS = COVID-19 career shock.

**Figure 2.**
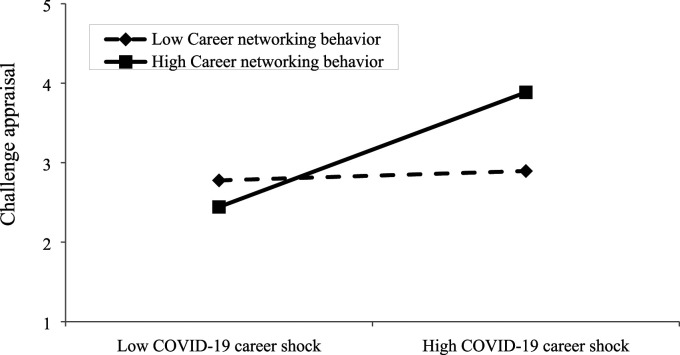
The moderating role of career networking behavior on the association between COVID-19 career shock and challenge appraisal.

**Figure 3.**
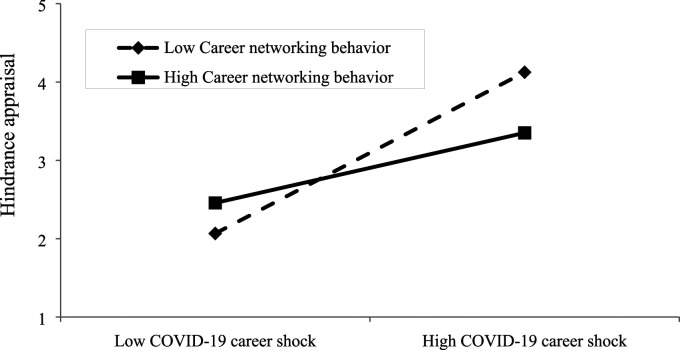
The moderating role of career networking behavior on the association between COVID-19 career shock and hindrance appraisal.

We used model 7 in PROCESS macro to analyse the moderated-mediation model. The results in Table 2 show that, as expected, the interaction term of career networking behavior with COVID-19 career shock on challenge appraisal (b = .331, SE = .060, *p* = .000) and hindrance appraisal (b = −.291, SE = .068, *p* = .001) were statistically significant with the expected sign. Figures 2 and 3 show the associations between COVID-19 career shock and challenge appraisal as well as hindrance appraisal at different levels of career networking behaviors, which further illustrate the nature of the interactions graphically. Simple slopes analysis shows that at the low level of career networking behavior, the relationship between COVID-19 career shock and challenge appraisal is weaker (b = .059, *p* = .000) than that at the high level (b = .721, *p* < .001) of career networking behavior. In addition, at the low level of career networking behavior, the relationship between COVID-19 career shock and hindrance appraisal is stronger (b = 1.029, *p* = .000) than that at the high level of career networking behavior (b = .447, *p* = .000). Altogether these results support Hypotheses 7 and 8.

Lastly, as shown in Table 3, the moderated-mediation index (Hayes, 2015) of career networking behavior on the indirect effect of COVID-19 career shock on self-perceived employability via challenge appraisal (index = .050, 95% CI = .158 to .627) and hindrance appraisal (index = −.032, 95% CI = .081 to .323) were also significant. At a lower level of career networking behavior, the indirect effect of COVID-19 career shock on self-perceived employability via challenge appraisal was .047, compared to .055 at a higher level of career networking behavior. The indirect effect of COVID-19 career shock on self-perceived employability via hindrance appraisal was −.031 at a lower level of career networking behavior compared to −.034 at a higher level of career networking behavior.

## Discussion

This study has applied the challenge-hindrance appraisal framework to contend that the overall implications of COVID-19 pandemic are the result of the interplay between students’ perception of COVID-19 pandemic as a threat as well as an opportunity for their employability. Based on multi-wave data collected with MBA students, the findings shed new insights on the self-perceived employability of senior university students, an important, yet neglected, segment of the future labor force in the context of COVID-19 career shock.

### Theoretical Contribution

The study makes several contributions to career research as follows. First, it adds to research on the employability of university students by highlighting the final year MBA students’ career shock experience caused by the COVID-19 pandemic (Akkermans et al., 2020). Research on career (e.g., Samuel et al., 2020) or university students during the pandemic has largely focused on the stressful and deplenting effects (e.g., Aucejo et al., 2020; Hite & McDonald, 2020; Wang et al., 2020) given the discruptive nature of the pandemic. However, our research contributes to the knowledge of how an unprecedented global crisis, like the COVID-19 pandemic, could present some positive challenges. This knowledge is important because it adds nuances to the conventional view that perceived employability is higher in times of economic prosperity than economic recession. While the conventional view highlights the changes in the demand (Berntson et al., 2006), the employability landscape shaped by the unprecedented changes caused by the COVID-19 pandemic are more complex than the quantity of supply and demand. Therefore, the dual pathway that the COVID-19 career shock could associate with one’s self-perceived employability provides a more complete picture in the career domain.

In this direction, the study enriches the employability research from a contextual view as well. Specifically, employability has been at the centre of many disciplines, especially during turbulent economic times (Byrne, 2022; Okay-Somerville & Scholarios, 2017). Multi-disciplinary research on self-perceived employability has noted the importance of the environment, suggesting that individuals form a coherent self-image about their possibilities of employment vis-à-vis changing labor market (Vanhercke et al., 2014). The findings here highlight not only the disruption in external factors but also agentic roles of individuals. In fact there are growing practice and voice for higher education to turn crisis into opportunity in response to COVID-19 (Yang & Huang, 2021). So our research provides research-informed evidence to support this increasing call.

Second, the study contributes to the research on employability by clarifying the underlying mechanism through which COVID-19 as a career shock associates with self-perceived employability. Prior work on employability has typically drawn from theories such as human/social capital theory (e.g., McArdle et al., 2007), career construction theory (e.g., Blokker et al., 2019), to identify factors that influence employability. Our work introduces an alternative theoretical framework, based on the transactional theory of stress, to explain how self-perceived employability can be shaped by one’s cognitive assessment of an external crisis. A key value of our theoretical framework lies in the explicit acknowledgement that the influence of career shock on self-perceived employability is not in an either-or fashion. The dual mediation pathway uncovered in our study provides empirical evidence that it can be appraised simultaneously as a challenge and a hindrance which in turn associates with self-perceived employability differently. An originality of the transactional theory of stress, as compared to other stress theories, is that it departs from treating the impact of the experienced exposure to a certain condition as uni-dimensional (Cavanaugh et al., 2000).

Investigating the appraisal process is important not just because it uncovers the black-box of the career shock – employability association. Also, it acknowledges the subjective interpretation of people who are exposed to these events (Searle & Auton, 2015). So rather than using a prior classification of stressors, the approach adopted in this study explains the cognitive processes that intervene between the encountered events and behavioral response. Indeed, prior studies have shown that work stressors (e.g., workload, responsibility, role conflict, role ambiguity (Webster et al., 2011) or job demands (e.g., time-pressure, task-complexity, and interruptions (Gerich & Weber, 2020) are positively correlated with challenge and hindrance appraisals. This study enriches this line of research to show that career shock also includes both appraisals simultaneously. Results of this research support our theorizing for departing from conventional approaches of treating the consequences of career shock events on employability from a unidimensional perspective and instead consider the simultaneous interplay between opportunities and threats that such event trigger.

Third, the study contributes to the career literature by adding a nuanced picture of the boundary condition regarding the relationship between career shock and self-perceived employability. Approaching the external labor market environment from a career shock perspective speaks to important psychological processes individuals engage and hence nicely captures the psycho-social nature of self-perceived employability. The finding about the moderating role of career networking behavior suggests that the cognitive appraisal process is contingent upon individuals’ behavior in gauging career-related resources. Research on cognitive appraisals has called for more studies to investigate how the challenge-hindrance framework works in different conditions, especially “identify[ing] factors that moderate the role of appraisals as transmitter of stressor effects, and, in the end, significantly impact how stressors influence outcomes” (LePine et al., 2016, p. 1037). The current study hence contributes to this line of research by introducing career networking behavior as the boundary condition that moderates the implications of career shock in the challenge-hindrance framework. The interesting finding is the critical role that career networking behavior can play in turning negative perception of employability from COVID-19 into positive perception.

Last but not least, this study is also a timely addition to the growing debate on graduate employability that increasing students’ capacity to reflect on experiences to become more self-aware and informed are more important than individual skills and competencies (Lees, 2002; Qenani et al., 2014). Understandings of how they perceive their employability in the wake of COVID-19 pandemic thus provide valuable insights for employers, students and their educational institutions on ways to strengthen the resilience of the future labor force in the face of future career shocks. Our study thus contributes to the line of research that enriches understandings of individuals’ capacity for adaptation in managing their employability (Heijde & Van Der Heijden, 2006; Melinde, 2014; Wittekind et al., 2010).

### Practical Implications

The findings of the study have several practical implications. First, the study highlights to educational institutes and future employers that university students perceive career shocks simultaneously as presenting both challenges and hindrances to their employability. Educational institutes could use this finding to design and offer career development programs that include training in skills that promote positive evaluations of challenging career situations. Future employers could better understand the needs and expectations of their potential employees and design their recruitment strategies that take into account the impact of career shock on employability. Additionally, the results show that the perception of COVID-19 pandemic as a hindrance (b = .738) was twice as much as presenting opportunities (b = .390). Educational institutes and employers could work together to provide more support and resources for individuals who struggle to find employment or maintain career during difficult times. Second, the finding related to the significant contribution of career networking behavior in influencing self-perceived employability of these MBA students sheds lights into career management strategies. For instance, it is advisable that career advisers at universities work towards providing students with psychological guidance in areas such as hope and resilience that have been shown to be an effective mechanism for positive thinking (Luthans et al., 2004). In addition, career counsellors should also offer career choice interventions to enhance students’ preparedness (Koivisto et al., 2011). Prior research shows that career choice interventions have on average a small to medium effect on key outcomes such as vocational identity, career maturity, career decidedness, and career decision-making self-efficacy, with self-efficacy perhaps most related to our study context. Moreover, coaching should be provided to university students to take control of their future employability through training programs that focuses on developing skills related to networking. Finally, another suggestion is that employers that rely on universities for their future talent pool can play an important role in presenting a positive image of how they tackle the external shock. Such positive reinforcement from the industry leaders and employers should increase students’ perceptions of external career shocks such as COVID-19 as presenting opportunities for skilled labor rather than being a threat to their future career.

### Limitation and Future Research Direction

We acknowledge there are limitations in the study. First, the sample is highly contextualized, which may not apply to students in other disciplines where online learning may not apply and where the industry is impacted by COVID differently. Future research could enlarge the sample and collect data across multiple countries. Second, we did not trace the changes in the perceived employability and cannot make causality claims. Future research could take a longitudinal design to explore whether or how employability perceptions changed. A longitudinal design will also contribute to understandings of temporal influences on students’ perception of their employability during career shock events.

## Conclusion

The lingering of the COVID-19 pandemic and the ongoing severe social and economic disruptions raise important questions about its impacts on the career plans of university students. We hope that the challenge-hindrance appraisal framework contributes to the career shock literature by providing new insights into university students’ career concerns as the COVID-19 pandemic unfolds.

## Note

1. We undertook a supplementary analysis by conducting structural equation modeling to test the hypotheses. The results are consistent with those obtained from SPSS PROCESS macro, which further adds confidence to our findings.
